# ARGscape: a modular, interactive tool for manipulation of spatiotemporal ancestral recombination graphs

**DOI:** 10.1093/bioinformatics/btag420

**Published:** 2026-06-25

**Authors:** Christopher A Talbot, Gideon S Bradburd

**Affiliations:** Department of Ecology and Evolutionary Biology, University of Michigan, Ann Arbor, MI 48109, United States; Department of Computational Biology, Cornell University, Ithaca, NY 14853, United States; Department of Ecology and Evolutionary Biology, University of Michigan, Ann Arbor, MI 48109, United States

## Abstract

**Summary:**

Ancestral recombination graphs (ARGs) are increasingly central to modern population genetics, yet ARG-based methods for spatiotemporal demographic inference remain underutilized in empirical settings due to fragmented workflows and a lack of exploratory tools. ARGscape addresses this by providing a unified framework, seamlessly integrating established and novel tools for ARG simulation, manipulation, and spatiotemporal inference into both graphical and command-line interfaces. ARGscape features dynamic 2- and 3-dimensional visualizations and a novel “spatial diff” visualization for quantitative comparison of ARG-based geographic inference methods. By integrating these various functionalities, ARGscape facilitates novel data exploration and hypothesis generation, bridging the gap between methods development and empirical adoption, and enabling educational uses.

**Availability and Implementation:**

ARGscape is available as a Python package on PyPI and as a live website for educational and simple demonstrative purposes at https://www.argscape.com. The source code and documentation are available on GitHub at https://github.com/chris-a-talbot/argscape.

## 1 Introduction

The study of genetic variation across time and space is fundamental to population genetics, providing insights into processes from disease outbreaks to species evolution (Beebe *et al*. 2013, Rehmann *et al*. 2025). The record of these processes can be read from the Ancestral Recombination Graph (ARG), which traces the complete genetic ancestry, including recombination and coalescent events, for a set of samples (Griffiths and Marjoram 1996, Rasmussen *et al*. 2014, Brandt *et al*. 2024, Lewanski *et al*. 2024, Nielsen *et al*. 2025). These structures can provide unprecedented clarity in studies of genetic variation across continuous space and time, moving beyond discrete populations and time points toward a more biologically realistic representation of evolution. Recent developments in ARG inference methods have made high-quality ARGs accessible for a wider range of datasets of varying size and quality (Kelleher *et al*. 2019, Speidel *et al*. 2019, Hubisz *et al*. 2020, Zhang *et al*. 2023, Deng *et al*. 2025). In particular, the succinct tree sequence structure has made the storage and analysis of very large ARGs computationally tractable (Kelleher *et al*. 2016, Ralph *et al*. 2020, Wong *et al*. 2024).

Consequently, ARGs are now central to a growing number of sophisticated inference methods (Hejase *et al*. 2022, Wohns *et al*. 2022, Tagami *et al*. 2024, Fan *et al*. 2025). In particular, a growing area of research is the use of ARGs to infer the spatial history of ancestors of a population (Wohns *et al*. 2022, Osmond and Coop 2024, Deraje *et al*. 2025, Grundler *et al*. 2025). However, despite their theoretical power, a broadly accessible and unified framework for interpreting ARGs as spatiotemporal objects remains elusive. This gap hinders their adoption by the broader community. While unified visualization and analysis of ARGs as spatiotemporal objects could promote broader adoption of these tools, phylogenetic tools like iTOL (Letunic and Bork 2024) and Taxonium (Sanderson 2022) are limited to tree structures, making them incompatible with reticulate graph-based ARGs. Tools like tsbrowse (Karthikeyan *et al*. 2025), tskit_arg_visualizer (Kitchens and Wong 2025), and Lorax (Katte and Corbett-Detig 2026) provide powerful interfaces for inspecting ARG topology, but they are not designed for the integrated spatiotemporal analysis and interactivity critical for robust exploration of many ecological and evolutionary questions. While tskit_arg_visualizer offers visualizations of ARGs with similar performance, it requires knowledge of Python to use. There is currently no platform for tskit-native, interactive spatiotemporal ARG analysis, visualization, and method comparison.

To bridge this gap and enhance the accessibility of ARGs, we present ARGscape: a tool for generating, exploring, manipulating, and analyzing ARGs as spatiotemporal records of evolutionary history. ARGscape integrates simulation, analysis, and visualization into a single, intuitive workflow, making complex analyses accessible to both researchers and students.

## 2 System and features

ARGscape is a full-stack web application with a Python-based backend and a React-based frontend, available as a Python package for local deployment, and includes a live demo online for smaller tasks. Its workflow is designed to be modular and intuitive, guiding the user from data input to visualization and export. The backend design is intended to facilitate rapid integration of new algorithms and features, ensuring that developments in the field can be incorporated and made accessible quickly. The ARGscape Python package also features a command-line interface incorporating core functionality without the need to load the full web application. As part of this work, we introduce FastGaia, a novel high-performance algorithm for spatial inference, and GaiaPy, a Python port of the Gaia algorithms to enhance the broader bioinformatics ecosystem (see Supplementary Data, available as supplementary data at *Bioinformatics* online).

### 2.1 Data input and simulation

Users can begin by either uploading existing tree sequences in tskit formats or by simulating new ones with integrated msprime tools (Kelleher *et al*. 2016, Nelson *et al*. 2020, Baumdicker *et al*. 2022). For command-line users, tree sequences can be loaded and stored using the argscape load command. Spatial coordinates for tree sequence nodes are extracted from the file when available, and may also be provided in .csv files. For demonstrative purposes, ARGscape uses the genealogical distance matrix to produce “toy” spatial coordinates for simulated samples.

### 2.2 Spatiotemporal inference

A core function of ARGscape is to provide a cohesive analytical environment that integrates multiple complex spatiotemporal inference tools, enabling direct comparison and hypothesis testing within a single interface. Once a tree sequence is loaded, we offer:

- *Statistics*, including heterozygosity, effective population size, and, when applicable, $F_{ST}$.
- *Temporal inference* using tsdate (Wohns *et al*. 2022).
- *Spatial inference* for ARGs with georeferenced samples. Users may choose from methods using local trees, including Wohns midpoint (heuristic midpoint on all trees, Wohns *et al*. (2022)); or those using the full ARG, including Gaia (heuristic maximum parsimony, Grundler *et al*. (2025)), FastGaia (parallelized heuristic maximum parsimony, see S3), or Sparg (probabilistic, Deraje *et al*. (2025)).

These analyses are initiated with simple button clicks in the user interface. The Python package also offers command-line tools for manipulating tree sequences, including spatiotemporal inference, using the argscape infer command. From command line, inference can be run on tree sequences of any size, limited only by each individual package’s abilities.

### 2.3 Interactive visualization

ARGscape offers multiple interactive visualization styles. Before visualizing, the user may select a subset of sample nodes, genomic or temporal regions to visualize. This is particularly useful for ARGs that extend deep into the past, for which visualizing only recent ancestry may be most informative. All visualizations are highly customizable, with toggleable labels and customizable colors and sizes. Mutation markers are placed along edges in proportion to their timing. Nodes, edges, and mutation markers include descriptive hover information for deeper exploration. Interactive sliders for genomic and temporal windows enable powerful data exploration, allowing users to isolate and visualize how specific ancestry segments have moved across time and, where applicable, space. A statistics panel shows summary statistics for the full ARG, as well as the currently selected genomic window, when applicable. Visualizations are size-limited due to browser constraints. While tools for visualizing larger ARGs are available in the app, ARGscape works best on ARGs with fewer than 2000 edges. ARGscape offers three visualization modes:

1)   *3D Spatial View:* For ARGs in which all nodes are georeferenced, a dynamic 3D plot rendered with deck.gl visualizes the ARG in geographic space, with time on the *z*-axis. This is best for exploring the geography of a population’s ancestors, both generally and for particular genomic regions (potentially those containing loci of interest). Coordinate reference systems (CRS) are automatically detected to display nodes on an appropriate unit grid or world map. Users can upload custom shapefiles for bespoke geographic contexts. Pairs of sample nodes that occur in identical locations and have consecutive node IDs are assumed to represent haplotypes from the same individual, and are therefore combined into a single node for clarity. This visualization mode opens the door to novel hypothesis generation and data exploration, including the visual assessment of coalescent rates over time and space. ARGscape 3D can render up to 2000 edges in under 20 seconds with 300MB of browser memory, with graphs of up to 50 000 edges being feasible.
2)   *3D Spatial “Diff” View:* A dynamic 3D plot rendered with deck.gl visualizes the difference in spatial locations on nodes in two fully-georeferenced ARGs with otherwise identical structure. Nodes are placed at the midpoint of their locations in each of two different ARGs, with a bar indicating the magnitude and direction of difference in locations between ARGs. This tool is unique to ARGscape and is highly useful for comparing inferred ancestral locations with simulated ground truth, including that output by SLiM. It is also useful for comparing across multiple inference methods to understand differences in the magnitude of error through time and space.
3)   *2D Non-spatial View:* A force-directed graph rendered using D3.js displays the topological structure of the ARG. This is best for exploring genealogical relationships, mutations, and coalescent and recombination event timing. Users may automatically arrange sample nodes using any one of seven ordering algorithms, including three designed specifically for tree sequences in ARGscape. Internal nodes are placed using a force simulation to ensure adequate spacing for visual clarity, but may also be moved with drag-and-drop functionality. The “dagre” mode places nodes using an edge-crossing minimization algorithm from the dagre-d3 React library. Users can interactively explore the graph structure by clicking nodes to view sub-ARGs or trace ancestral lineages. ARGscape 2D can render up to 2000 edges in under two minutes with 300 MB of browser memory, with larger graphs becoming intractable.

### 2.4 Accessibility and data export

ARGscape is available as both a public web application and as a Python package. The web application limits memory and CPU usage, making it ideal for quick tasks like educational demonstrations or small-scale testing. The Python package uses local resources, enabling resource-intensive use cases and integration into local pipelines. Users can download modified tree sequences and intermediate data from inference methods for downstream analysis. Publication-quality images of any visualization can also be exported, facilitating integration into manuscripts, presentations, and educational materials. We note that biobank-scale ARGs exceed the practical rendering limits of our visualization frameworks; users working at this scale should consider Lorax (Katte and Corbett-Detig 2026).

## 3 Conclusion

ARGscape provides a much-needed, user-friendly platform that integrates the simulation, analysis, and spatiotemporal exploration of ancestral recombination graphs. By integrating both established and novel inference tools in an intuitive interface alongside novel interactive visualizations, it significantly lowers the barrier to entry for researchers and students interested in exploring the full richness of ARGs. ARGscape simplifies the process of asking and answering questions about how ancestry unfolds across the genome over time and across geographic space. Future directions will focus on enhancing performance for biobank-scale datasets and expanding the tool’s analytical capabilities to visualize key demographic processes through time, including migration and admixture. We also aim to further develop its utility as an interactive learning platform for population genetics education by incorporating guided tutorials and visual-based lessons. By providing novel algorithms, a unique analytical visualization for methods comparison, and an intuitive workflow, we believe ARGscape is an essential platform for population genetic research that will grow alongside the field.

**Figure 1:**
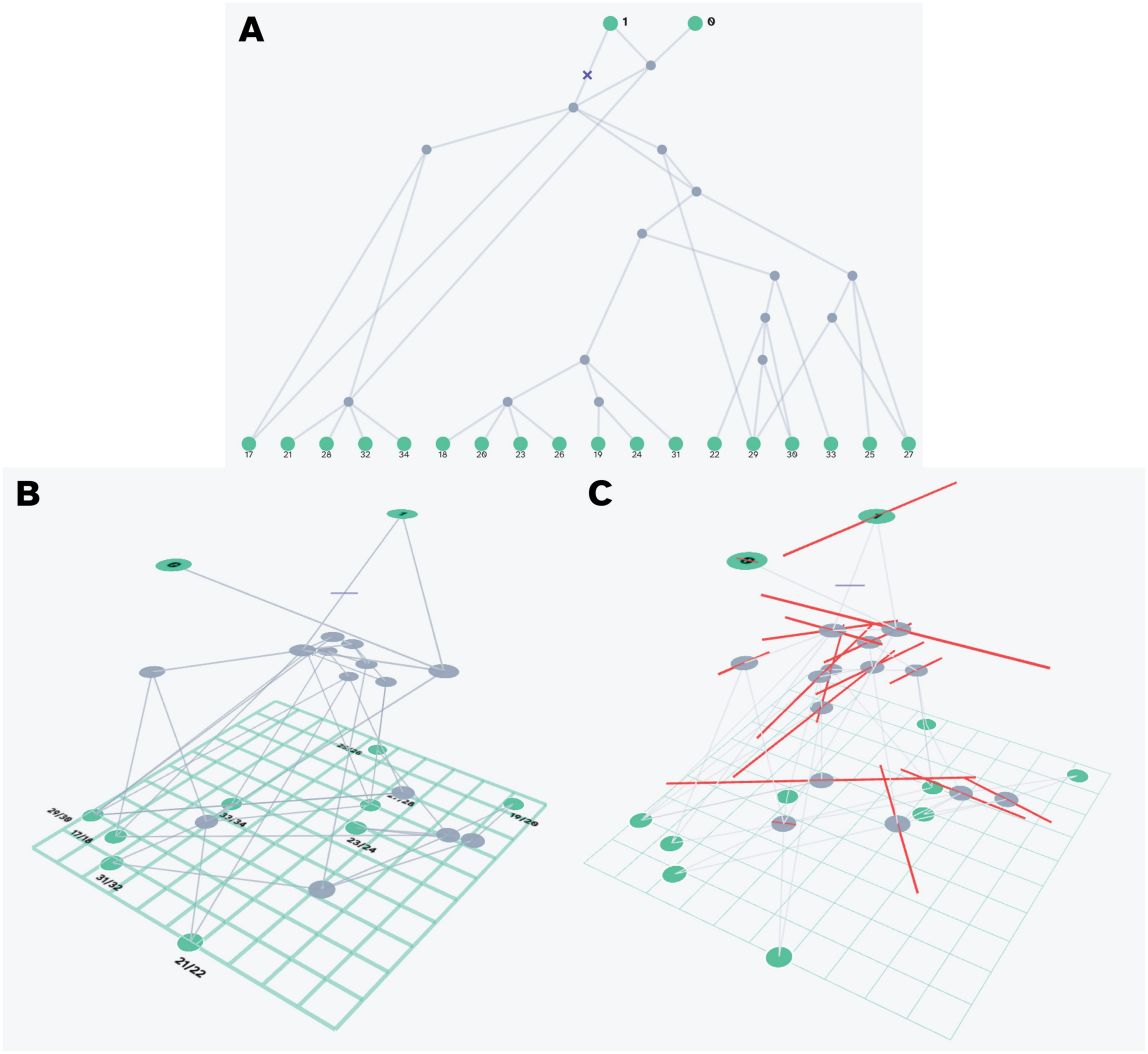
Visualizations of an ancestral recombination graph (ARG) generated by a spatially explicit evolutionary simulation in SLiM 5.1. (A) A 2D visualization, displaying the topology of the ARG. Our ‘consensus’ node ordering generates this layout. Green nodes represent samples and roots, and gray nodes represent ancestral nodes. Branches with mutations along them are marked with a red “x.” In ARGscape, node locations may be adjusted, node and edge labels toggled, and colors modified. (B) A 3D visualization of the same ARG with ground truth spatial data from the simulation. Branches with mutations along them are marked with blue bars. Generations are evenly spaced along the *z*-axis. Sample nodes from the same individual are labeled together because they originate from the same location. In ARGscape, spacing may be adjusted, node and edge labels toggled, and colors modified. Nodes may be selected to display parent or child subARGs. (C) A 3D “spatial diff” visualization. Ancestral locations along the same ARG were re-inferred using the Gaia quadratic algorithm in ARGscape. Nodes are placed at the midpoint of the simulated and inferred locations, with red bars connecting the ground truth and inferred locations. This can be used to visualize the quantity and directionality of error in an inference method against ground truth. Hovering over nodes reveals metadata, including their geographic locations in each version of the ARG, and the distance between them.

## Supplementary Material

btag420_Supplementary_Data

## Data Availability

Source code and documentation are available at https://github.com/chris-a-talbot/argscape. Figure 1 uses a tree sequence generated by a simulation in SLiM v5.1 (Haller *et al*. 2026). The tree sequence and SLiM simulation script used to generate visualizations for Figure 1 are available on Zenodo under doi.org/10.5281/zenodo.20090220. ARGscape v0.7.5 is archived on Zenodo at doi.org/10.5281/zenodo.20077950.
