# Supplementary material for: ARGscape: a modular, interactive tool for manipulation of spatiotemporal ancestral recombination graphs
Source: Bioinformatics. 2026 Jun 25;42(7):btag420. doi: 10.1093/bioinformatics/btag420 (PMC13371762; doi:10.1093/bioinformatics/btag420)
Supplement: btag420_Supplementary_Data [file btag420_supplementary_data.pdf]

Supplementary Information

`ARGscape`: A modular, interactive tool for manipulation of spatiotemporal ancestral recombination graphs

Christopher A. Talbot[1,2,*] and Gideon S. Bradburd[1]

[1]Department of Ecology and Evolutionary Biology, University of Michigan, Ann Arbor, MI, USA
[2]Department of Computational Biology, Cornell University, Ithaca, NY, USA

Contents

S1 Installation & Usage 2
S1.1 Using the Hosted Web Application . . . 2
S1.2 Installing `ARGscape` Locally . . . 2
S1.3 Running the Web Application Locally . . . 2
S1.4 Using the Command-Line Tools . . . 2
S1.4.1 argscape serve . . . 2
S1.4.2 argscape load . . . 3
S1.4.3 argscape infer . . . 3
S1.4.4 argscape viz . . . 3
S1.5 Using the Python API . . . 3

S2 Comparison of ARG and Phylogenetic Visualization Tools 4

S3 `FastGaia` 6
S3.1 `FastGaia` Algorithm Notation and Definitions . . . 6
S3.2 `FastGaia` Algorithm S1: Continuous Location Inference . . . 7
S3.3 `FastGaia` Algorithm S2: Discrete State Inference . . . 8
S3.4 Example: Discrete geographic inference with FastGaia . . . 9
S3.4.1 Toy example . . . 9
S3.4.2 Comparison to gaia . . . 10

S4 `GaiaPy` 11

S5 2D Visualization Tip Ordering Algorithms 11
S5.1 Numeric . . . 11
S5.2 First Tree . . . 12
S5.3 Center Tree . . . 12
S5.4 Consensus . . . 12
S5.5 Ancestral Path . . . 12
S5.6 Coalescence . . . 12
S5.7 Dagre-d3 . . . 12

S1 Installation & Usage

S1.1 Using the Hosted Web Application

The latest production version of `ARGscape` is available at `https://www.argscape.com/`. The interactive interface guides users through the various modes of use and functionality, including file management, simulation, visualization, inference, and output downloading. Files stored on the hosted web server will be stored privately and securely for up to 24 hours. The web server storage is subject to being cleared, without notice, during updates.

We recommend using the hosted application only for **educational** and/or **exploratory** purposes due to the potential for data loss and the limited computational capabilities of our web server. Users are limited to a small amount of computing power for simulation, inference, and visualization, which will be insufficient for most full-scale projects.

S1.2 Installing `ARGscape` Locally

The latest production version of `ARGscape` is available as a Python package hosted on the Python Package Index (PyPI). To install, users must have Python version 3.8 or later. Then, from a terminal in which Python is available, users should run the following commands:

```
> python -m pip install --upgrade pip
> python -m pip install argscape
```

`ARGscape` will be installed in the Python environment.

S1.3 Running the Web Application Locally

To run the web application hosted on a local machine, first see Supplemental Information 1.2 – Installing `ARGscape` Locally. Once `ARGscape` is installed, the web application can be started from any terminal in which Python is available and `ARGscape` is installed using the following command:

```
> argscape serve
```

This command will launch the complete `ARGscape` web application in a browser window, hosted on your local machine. For additional options, including customizing the port `ARGscape` is hosted on and disabling certain features, run:

```
> argscape serve --help
```

S1.4 Using the Command-Line Tools

All command-line tools are accessed as subcommands of the `argscape` command. Once `ARGscape` is installed (see S1.2), the available subcommands include:

S1.4.1 argscape serve

The `argscape serve` command launches the web application in a browser window.

S1.4.2 argscape load

The argscape load command includes features for session file management, including loading tree sequences from files using

> argscape load load --file <filename>

or, with sample and/or node locations, using

> argscape load load-with-locations --file <filename> --sample-csv <filename> --node-csv <filename>

To view the complete set of file management commands, run

> argscape load --help

S1.4.3 argscape infer

The argscape infer command unifies features for running spatial and temporal inference methods on tree sequences. A guided interactive interface is provided by running

> argscape infer

Inference can also be run non-interactively:

> argscape infer run --input <filename> --method fastgaia --output <directory>

To view the complete set of spatiotemporal inference commands, run

> argscape infer --help

S1.4.4 argscape viz

The argscape viz command generates standalone visualizations directly from a tree sequence file, without launching the full web application. By default, the visualization opens in a browser window:

> argscape viz myfile.trees

To export a static image instead, provide an output path:

> argscape viz myfile.trees -o output.png

Common options include --mode (force_graph or spatial_3d), --theme, --max-samples, --genomic-range, and --show-mutations. For the full set of options, run

> argscape viz --help

S1.5 Using the Python API

ARGscape provides a Python API for programmatic visualization and inference, suitable for scripting and integration with Jupyter notebooks. After installing ARGscape (see S1.2), the core workflow is:

```python
import argscape

# Visualize a tree sequence
viz = argscape.visualize(ts, theme="liquid", show_mutations=True)
viz.show()            # Open in browser
viz.display()         # Inline in Jupyter
viz.export("fig.png")  # Static export (requires Playwright)
```

```
# Run spatial inference, then visualize the result
result = argscape.infer(ts, method="fastgaia")
argscape.visualize(result.ts, mode="spatial_3d").show()
```

The `visualize()` function accepts options for subsetting (`max_samples`, `genomic_range`), layout (`sample_order`, `temporal_spacing`), node and edge styling, and animation. The `infer()` function supports several spatial methods (`midpoint`, `fastgaia`, `gaia-quadratic`, `gaia-linear`) and temporal inference via `tsdate`. For a complete list of parameters, see the package documentation or call `help(argscape.visualize)` and `help(argscape.infer)`.

S2 Comparison of ARG and Phylogenetic Visualization Tools

To contextualize `ARGscape` within the broader ecosystem of genealogical and phylogenetic visualization tools, we provide a comprehensive comparison in Table S1. This table compares features across ARG-specific tools, phylogenetic visualization platforms, and `ARGscape`.

Table S1: Comparison of ARG and phylogenetic visualization tools. Features are marked as supported (✓), not supported (–), or partially supported (∼).

**Feature**	**ARGscape**	**tsbrowse**	**tskit_arg_visualizer**	**Lorax**	**iTOL**	**Taxonium**	**ggtree**
Data Types							
ARG / Tree Sequence Support	✓	✓	✓	✓	–	–	–
Phylogenetic Tree Support	–	–	–	–	✓	✓	✓
Newick/Nexus Import	–	–	–	–	✓	✓	✓
Visualization Capabilities							
2D Topology View	✓	–	✓	✓	✓	✓	✓
3D Spatial/Geographic View	✓	–	–	–	–	–	–
Temporal Axis	✓	✓	✓	✓	–	∼	✓
Mutation Visualization	✓	✓	∼	✓	–	✓	✓
Method Comparison (Diff View)	✓	–	–	–	–	–	–
Interactivity							
Interactive Pan/Zoom	✓	✓	∼	✓	✓	✓	–
Node Selection/Filtering	✓	∼	∼	✓	✓	✓	∼
Genomic Window Sliding	✓	✓	∼	✓	–	–	–
Temporal Window Sliding	✓	∼	–	✓	–	–	–
Customizable Styling	✓	∼	✓	∼	✓	∼	✓
Animation Support	✓	–	–	–	–	–	–
Integrated Analysis							
Temporal Inference	✓	–	–	–	–	–	–
Spatial Inference	✓	–	–	–	–	–	–
Simulation	✓	–	–	–	–	–	–
Summary Statistics	✓	✓	–	–	–	∼	–
Accessibility							
Web Interface	✓	✓	–	✓	✓	✓	–
Local Installation	✓	✓	✓	✓	–	✓	✓
Command-Line Tools	✓	✓	✓	✓	–	✓	–
Python API	✓	∼	✓	–	∼	∼	–
R API	–	–	–	–	∼	–	✓
Scalability							
Max Sample Size	Moderate	Very Large	Small	Large	Large	Very Large	Moderate
Max Edges (approx.)	∼50K (3D only)	Millions	∼1K	–	–	–	–
Clustering for Large Graphs	✓	∼	–	–	∼	∼	∼
Export							
Image Export	✓	✓	✓	–	✓	–	✓
Data Export	✓	–	✓	–	✓	∼	✓

Tools: tsbrowse [Karthikeyan et al., 2025]; tskit_arg_visualizer [Kitchens & Wong, 2025]; Lorax [Katte & Corbett-Detig, 2026]; iTOL [Letunic & Bork, 2024]; Taxonium [Sanderson, 2022]; ggtree [Yu et al., 2017].

S3 `FastGaia`

`FastGaia` is a set of algorithms for inferring the geographic locations of ancestral nodes in a tree sequence given georeferenced samples. Drawing inspiration from `Gaia` and Wohns' midpoint approach [Wohns et al., 2022, Grundler et al., 2025], this approach aims to utilize more of the information encoded in the ARG than a simple midpoint approach, while running faster than `Gaia` by implementing a parallelizable greedy algorithm approach. The ease of incorporating `FastGaia` within the `ARGscape` framework demonstrates the flexibility of integrating new methods into `ARGscape`'s modular framework.

Like `Gaia`, `FastGaia` can operate in continuous space (no barriers to dispersal, Euclidean cost function) or discrete space (using a uniform transition cost between states or an input transition cost matrix). Note that `ARGscape` uses only the continuous-space version of the algorithm. The complete details of both `FastGaia` algorithms are provided below, along with the necessary notation.

The latest stable version of `FastGaia` can be installed in Python environments running Python version 3.8 or later by running the command `pip install fastgaia`. The code and development versions can be found on GitHub at `https://github.com/chris-a-talbot/fastgaia`.

S3.1 `FastGaia` Algorithm Notation and Definitions

- T: Tree sequence with nodes V and edges E
- $n = |V|$: Number of nodes
- $t(u)$: Time (age) of node u
- $S \subset V$: Set of sample nodes with known locations/states
- $E(u)$: Set of edges where u is the parent
- $C(u) = \{v : (u, v) \in E\}$: Set of children of node u
- $P(u) = \{w : (w, u) \in E\}$: Set of parents of node u
- For edge $e = (u, v)$:
  - $s(e)$: Genomic span (right - left coordinates)
  - $b(e) = t(u) - t(v)$: Branch length (temporal distance)
- $\mathcal{E}_u$: Set of valid edges from parent u to children with known locations/states (used locally)
- ω_e: Weight assigned to edge e (continuous inference)

S3.2 `FastGaia` Algorithm S1: Continuous Location Inference

Input:

- Tree sequence $T = (V, E)$
- Sample locations $\mathcal{L}_S = \{\ell_u \in \mathbb{R}^d : u \in S\}$
- Boolean flags: w_{span}, w_{branch}

Output:

- Inferred locations $\mathcal{L} = \{\ell_u \in \mathbb{R}^d : u \in V\}$

Algorithm S1: Continuous Location Inference

Initialize $\ell_u \leftarrow \text{NaN} \in \mathbb{R}^d$ for all $u \in V$
$\ell_u \leftarrow \mathcal{L}_S(u)$ for all $u \in S$ ▷ Assign known sample locations
Partition nodes: $V_\tau = \{u \in V : t(u) = \tau\}$ for each unique time τ
Sort times: $\mathcal{T} = \{\tau_1, \tau_2, \dots, \tau_k\}$ where $\tau_1 < \tau_2 < \cdots < \tau_k$
for each time $\tau \in \mathcal{T}$ **do** ▷ Process from present to past
    for each node $u \in V_\tau$ **do** ▷ Parallel processing possible
        if $u \in S$ **then**
            continue ▷ Sample location already known
        end if
        $\mathcal{E}_u \leftarrow \{e = (u, v) : v \in C(u) \text{ and } \ell_v \neq \text{NaN}\}$
        if $\mathcal{E}_u = \emptyset$ **then**
            $\ell_u \leftarrow \text{NaN}$ ▷ No valid children
            continue
        end if
▷ Compute weighted average location
        for each edge $e = (u, v) \in \mathcal{E}_u$ **do**
            $\omega_e \leftarrow 1.0$
            if $w_{\text{span}} = \text{True}$ **then**
                $\omega_e \leftarrow s(e)$
            end if
            if $w_{\text{branch}} = \text{True}$ **then**
                $\omega_e \leftarrow \omega_e \cdot \frac{1}{b(e)}$ ▷ Inverse branch length
            end if
        end for
        $W \leftarrow \sum_{e \in \mathcal{E}_u} \omega_e$
        if $W = 0$ **then**
            $\ell_u \leftarrow \frac{1}{|\mathcal{E}_u|} \sum_{e=(u,v) \in \mathcal{E}_u} \ell_v$ ▷ Equal weights fallback
        else
            $\ell_u \leftarrow \frac{1}{W} \sum_{e=(u,v) \in \mathcal{E}_u} \omega_e \cdot \ell_v$ ▷ Weighted average location of children
        end if
    end for

end for
return $\mathcal{L} = \{\ell_u : u \in V\}$

Weight Formula: For edge $e = (u, v)$ connecting parent u to child v:

$$\omega_e = \begin{cases} s(e) \cdot \frac{1}{b(e)} & \text{if } w_{\text{span}} \wedge w_{\text{branch}} \\ s(e) & \text{if } w_{\text{span}} \wedge \neg w_{\text{branch}} \\ \frac{1}{b(e)} & \text{if } \neg w_{\text{span}} \wedge w_{\text{branch}} \\ 1 & \text{if } \neg w_{\text{span}} \wedge \neg w_{\text{branch}} \end{cases}$$

Location Update:

$$\ell_u = \frac{\sum_{v \in C(u)} \omega_{(u,v)} \cdot \ell_v}{\sum_{v \in C(u)} \omega_{(u,v)}}$$

S3.3 `FastGaia` Algorithm S2: Discrete State Inference

Input:

- Tree sequence $T = (V, E)$
- Sample states $\mathcal{S}_S = \{\sigma_u \in \{1, 2, \ldots, m\} : u \in S\}$
- Cost matrix $M \in \mathbb{R}^{m \times m}$ (optional): M_{ij} = cost of transition from state i to state j

Output:

- Inferred states $\mathcal{S} = \{\sigma_u \subseteq \{1, \ldots, m\} : u \in V\}$ (set-valued to account for ties)

Algorithm S2: Discrete State Inference

Initialize $\sigma_u \leftarrow \emptyset$ for all $u \in V$
$\sigma_u \leftarrow \{\mathcal{S}_S(u)\}$ for all $u \in S$ ▷ Assign known sample states
Determine state space: $\Sigma = \{1, 2, \ldots, m\}$
Partition nodes: $V_\tau = \{u \in V : t(u) = \tau\}$ for each unique time τ
Sort times: $\mathcal{T} = \{\tau_1, \tau_2, \ldots, \tau_k\}$ where $\tau_1 < \tau_2 < \cdots < \tau_k$
for each time $\tau \in \mathcal{T}$ **do** ▷ Process from leaves to root
    for each node $u \in V_\tau$ **do** ▷ Parallel processing possible
        if $u \in S$ **then**
            continue ▷ Sample state already known
        end if
        ▷ Accumulate cost for every candidate state
        for each candidate state $\alpha \in \Sigma$ **do**
            $\mathcal{C}(u, \alpha) \leftarrow 0$
            for each edge $e = (u, v)$ where $v \in C(u)$ and $\sigma_v \neq \emptyset$ **do**
                for each state $\beta \in \sigma_v$ **do**
                    $c \leftarrow M_{\alpha,\beta}$ if M provided, else $c \leftarrow 1$

$\mathcal{C}(u, \alpha) \leftarrow \mathcal{C}(u, \alpha) + c \cdot s(e) \cdot b(e)$
end for
end for
end for
$c^* \leftarrow \min_{\alpha \in \Sigma} \mathcal{C}(u, \alpha)$
$\sigma_u \leftarrow \{\alpha \in \Sigma : \mathcal{C}(u, \alpha) = c^*\}$ ▷ All optimal states
end for
end for
return $\mathcal{S} = \{\sigma_u : u \in V\}$

Cost Formula: For node u and candidate state α:

$$\mathcal{C}(u, \alpha) = \sum_{v \in C(u)} \sum_{\beta \in \sigma_v} c(\alpha, \beta) \cdot s_{uv} \cdot b_{uv}$$

where the transition cost function is

$$c(i, j) = \begin{cases} M_{ij}, & \text{if a cost matrix } M \text{ is provided} \\ 1, & \text{otherwise (uniform cost)} \end{cases}$$

State Assignment:

$$\sigma_u = \arg\min_{\alpha \in \Sigma} \mathcal{C}(u, \alpha)$$

Note: σ_u may include multiple states if there are ties for the minimum cost.

S3.4 Example: Discrete geographic inference with FastGaia

In addition to continuous spatial inference, FastGaia supports *discrete state inference*, in which each node in the ARG is assigned to one of K categorical geographic states (e.g., named populations, islands, or habitat types) rather than a point in continuous space. This mode is useful when samples are associated with discrete populations or regions and the research question concerns ancestral population membership rather than precise coordinates. Below, we describe the discrete inference algorithm, demonstrate it on a toy example, and compare it to the original *gaia* implementation.

A key distinction from continuous FastGaia is the weighting scheme. In continuous mode, branch lengths are inverse-weighted (shorter branches contribute more), reflecting the expectation that closely related nodes are geographically proximate. In discrete mode, the transition cost is instead *directly* weighted by the product of span and branch length: longer edges spanning more of the genome contribute more to the total cost, penalizing state transitions proportionally to their evolutionary and genomic extent.

S3.4.1 Toy example

To illustrate how the cost matrix influences discrete inference, we present a toy example on a 3×3 grid of discrete locations (Figure S1). Ten samples are unevenly distributed across the grid, with 7 on the western side (columns 0–1) and 3 on the eastern side (column

2). An asymmetric cost matrix encodes a geographic barrier between columns 1 and 2: crossing eastward costs 4 per step, while all other adjacent transitions (westward, northward, southward) cost 1 per step.

Setup. Each sample is assigned to one of 9 grid cells as its discrete state, and the 9×9 cost matrix is derived from the step-cost structure described above. The inference is run via the command line or Python API:

```
fastgaia --tree example.trees \
         --discrete-sample-locations samples.csv \
         --cost-matrix costs.csv \
         --output-inferred-discrete inferred_states.csv
```

Inference walkthrough. Nodes are processed bottom-up by time. At early times, ancestors of samples within the same grid cell are trivially assigned to that cell (e.g., the ancestor of three samples at $(0, 0)$ is placed at $(0, 0)$).

The cost matrix becomes influential when ancestors are shared between western and eastern samples. Consider an internal node at $t = 3$ whose children are at cells $(1, 0)$ and $(2, 0)$:

$$\text{Cost}(u, (1, 0)) = C_{(1,0),(1,0)} \cdot w_1 + C_{(1,0),(2,0)} \cdot w_2 = 0 + 4w_2 \tag{1}$$

$$\text{Cost}(u, (2, 0)) = C_{(2,0),(1,0)} \cdot w_1 + C_{(2,0),(2,0)} \cdot w_2 = 1 \cdot w_1 + 0 \tag{2}$$

With equal weights, cell $(2, 0)$ (cost $= w$) is preferred over $(1, 0)$ (cost $= 4w$). The asymmetry arises because placing the ancestor on the western side of the barrier requires its eastern child to have crossed eastward—an expensive transition. The algorithm therefore places the ancestor on the eastern side, where the child's westward crossing is cheap.

This effect compounds at deeper time levels. Despite the majority of samples (7 of 10) being located on the western side of the grid, the root of the tree is inferred at cell $(2, 1)$—on the eastern side of the barrier. This result demonstrates how asymmetric transition costs can override sample density, producing ancestral placements that reflect the directionality of geographic barriers rather than simply the spatial distribution of descendants.

S3.4.2 Comparison to gaia

The original *gaia* method (Grundler et al.) performs discrete ancestral state reconstruction using a maximum parsimony framework that operates on each local tree independently. For each local tree in the tree sequence, *gaia* applies a Fitch-like downpass–uppass algorithm to assign states to internal nodes, then aggregates results across trees weighted by their genomic span.

Discrete FastGaia differs in two key respects:

1. **ARG-level operation.** Rather than iterating over individual local trees, FastGaia operates directly on the full edge table of the ARG. Each edge contributes to the cost calculation for its parent node in proportion to its genomic span and branch length.

This avoids redundant computation when the same parent–child relationship appears across many local trees with identical topology, yielding substantial speedups for large tree sequences.

2. **Single-pass inference.** FastGaia processes nodes in a single bottom-up pass ordered by node time, assigning states greedily. In contrast, *gaia*'s Fitch algorithm uses a two-pass approach (downpass to compute state sets, uppass to resolve ambiguities) on each local tree. The single-pass approach in FastGaia is faster but may produce more ties and does not benefit from the uppass refinement step.

As noted in the FastGaia documentation, the accuracy of discrete state inference has not been formally benchmarked against *gaia*. For continuous inference, FastGaia infers coordinates within 0.16 units of the true location on a 20×20 plane (compared to 0.15 for *gaia*), with accuracy being highest for recent ancestors and decreasing for deeper nodes. We expect a qualitatively similar accuracy–speed tradeoff for discrete inference: FastGaia should closely match *gaia* for nodes with many nearby descendants but may diverge for deep ancestors with sparse descendant information. We demonstrate a comparison of Gaia (quadratic) and FastGaia in Figure S2, applied to a subset of the tree sequence of human chromosome 18. We further compare FastGaia to Wohns midpoint on simulated data in Figure S3.

S4 `GaiaPy`

As part of our effort to incorporate a wide range of ARG-based spatial demographic inference tools within the `ARGscape` platform, we introduce `GaiaPy`, a Python package incorporating the continuous-space algorithms from the `Gaia` package [Grundler et al., 2025].

The latest stable version of `GaiaPy` can be installed in Python environments running Python version 3.8 or later by running the command `pip install geoancestry`. The code, documentation, and development versions can be found on GitHub at `https://github.com/chris-a-talbot/gaiapy`.

S5 2D Visualization Tip Ordering Algorithms

While all 2D `ARGscape` visualizations are fully interactive and customizable, we also provide a diverse set of tree sequence-specific tip ordering algorithms designed to clarify complex, tangled graph layouts. The available tip ordering algorithms are detailed below. Ideal choice of tip ordering algorithm will vary on a case-by-case basis, with no clear general rules for which to choose in what scenario. However, especially for very complex graphs, `dagre-d3` mode will often produce the clearest graphs.

S5.1 Numeric

The numeric tip ordering algorithm places sample nodes along the x-axis in order of increasing node ID. Ancestral nodes are placed using a force-directed simulation. This ordering will often result in complex and tangled visualizations.

S5.2 First Tree

The first-tree tip ordering algorithm places sample nodes along the x-axis in the order of a minlex postorder traversal of the first local tree in the tree sequence. This order is generated automatically by `tskit` [Kelleher et al., 2016, Ralph et al., 2020, Wong et al., 2024], and is the same ordering algorithm used by `tskit_arg_visualizer` [Kitchens & Wong, 2025].

S5.3 Center Tree

The center-tree tip ordering algorithm places sample nodes along the x-axis in the order of a minlex postorder traversal of the middle local tree in the tree sequence. If only one local tree is available, this is the same order as in first-tree. If two trees meet the "center" criteria, the first is used.

S5.4 Consensus

The consensus tip ordering algorithm orders sample nodes along the x-axis according to a majority vote by minlex postorder traversals across K local trees in the tree sequence, where K scales with number of local trees, and is bounded by $[1, 50]$. The K local trees are selected from evenly spaced genomic positions across the tree sequence. If only one local tree is available, this is the same order as in first-tree or center-tree.

S5.5 Ancestral Path

The ancestral path tip ordering algorithm aims to group sample nodes by shared ancestry and similar time to coalescence. It creates groups of samples that coalesced at similar times, then places groups with more recent shared ancestry closer to the center of the graph. This aims to minimize path crossings by putting groups with deeper ancestry – and therefore longer edges – towards the outside of the graph.

S5.6 Coalescence

The coalescence tip ordering algorithm orders sample nodes along the x-axis in decreasing order of time to coalescence.

S5.7 Dagre-d3

When `dagre-d3` mode is enabled, all nodes in the graph are placed by the `dagre-d3` React library using an edge-crossing minimization algorithm. The force-directed simulation is disabled when this mode is active.

References

Grundler, MC, Terhorst, J, Bradburd, GS. A geographic history of human genetic ancestry. *Science* 2025; 387: 1391–1397.

Katte, P, Corbett-Detig, R. Interactive exploration of biobank-scale ancestral recombination graphs with Lorax. *bioRxiv* 2026; doi: 10.64898/2026.02.19.706861.

Karthikeyan, S, Jeffery, B, Mbuli-Robertson, D, et al. Tsbrowse: An interactive browser for Ancestral Recombination Graphs. *Bioinformatics* 2025; 41: btaf393.

Kelleher, J, Etheridge, AM, McVean, G. Efficient coalescent simulation and genealogical analysis for large sample sizes. *PLoS Comput Biol* 2016; 12: e1004842.

Kitchens, J, Wong, Y. tskit_arg_visualizer: interactive plotting of ancestral recombination graphs. *Bioinform Adv* 2025; 5: vbaf302.

Letunic, I, Bork, P. Interactive Tree of Life (iTOL) v6: recent updates to the phylogenetic tree display and annotation tool. *Nucleic Acids Res* 2024; 52: W78–W82.

Ralph, P, Thornton, K, Kelleher, J. Efficiently summarizing relationships in large samples: A general duality between statistics of genealogies and genomes. *Genetics* 2020; 215: 779–797.

Sanderson, T. Taxonium, a web-based tool for exploring large phylogenetic trees. *eLife* 2022; doi: 10.7554/eLife.82392.

Wohns, AW, Wong, Y, Jeffery, B, et al. A unified genealogy of modern and ancient genomes. *Science* 2022; 375: eabi8264.

Wong, Y, Ignatieva, A, Koskela, J, et al. A general and efficient representation of Ancestral Recombination Graphs. *Genetics* 2024; 228: iyae100.

Yu, G, Smith, DK, Zhu, H, Guan, Y, Lam, TT. ggtree: an R package for visualization and annotation of phylogenetic trees with their covariates and other associated data. *Methods Ecol Evol* 2017; 8: 28–36.

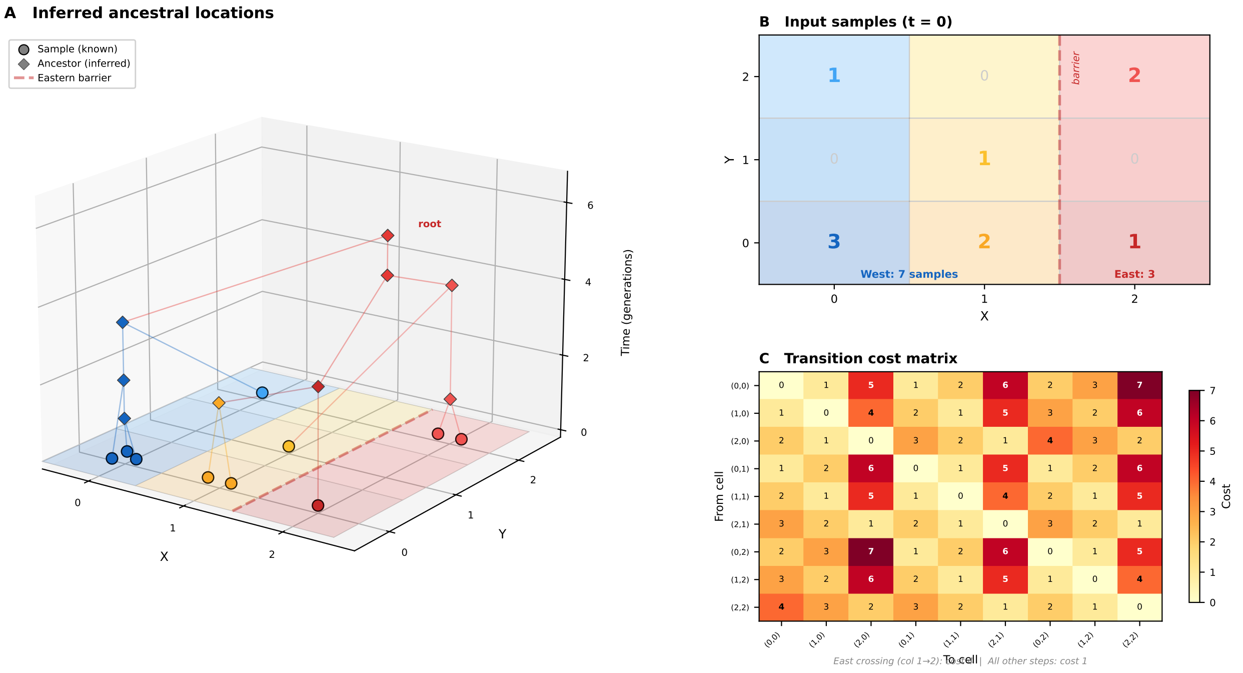

Figure S1: Discrete FastGaia applied to a toy example on a 3×3 grid with an eastern barrier. **(A)** 3D spatial-temporal view of the inferred ARG. The XY plane represents the grid (colored cells on the floor), and the vertical axis represents time. Sample nodes (circles) sit at $t = 0$; inferred ancestors (diamonds) are positioned at their inferred grid cell and time. Despite most samples being on the western side (blue), ancestors progressively shift eastward (red) at deeper times due to the barrier's asymmetric cost. The dashed red line marks the barrier between columns 1 and 2. **(B)** Distribution of the 10 input samples across the grid. **(C)** The 9×9 transition cost matrix. Eastward crossings from column 1 to column 2 incur a cost of 4 per step; all other adjacent transitions cost 1. The asymmetry is visible as the off-diagonal band of high costs.

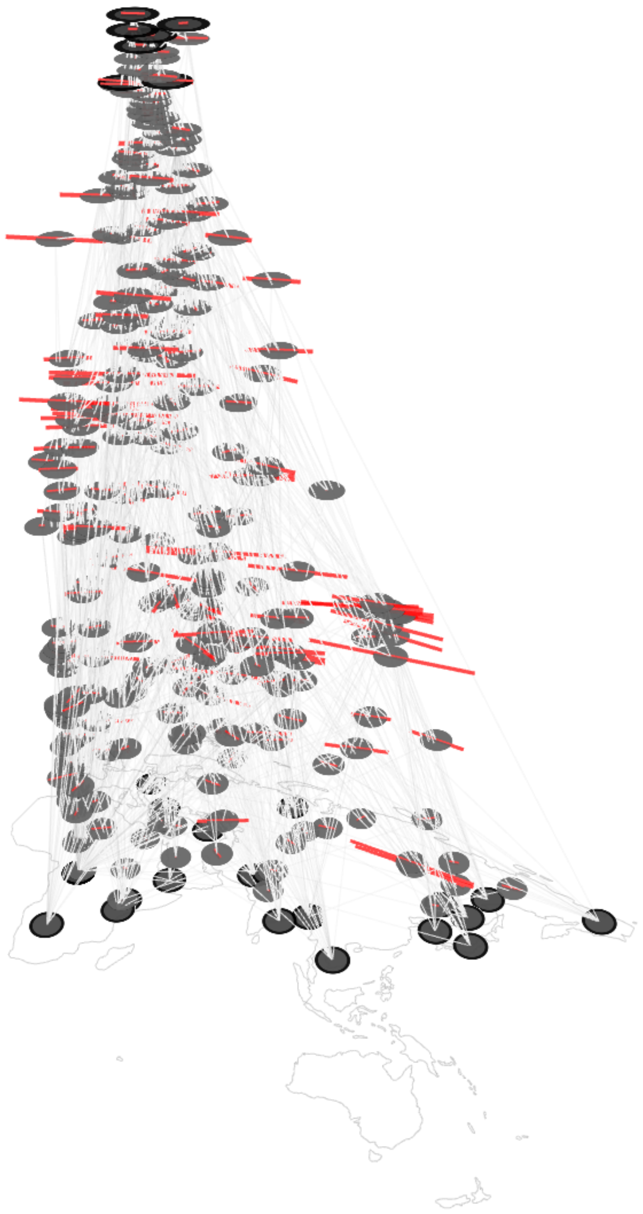

Figure S2: A ‘spatial diff’ view, comparing location inference between continuous FastGaia and Gaia quadratic algorithms, applied to a simplified tree sequence of 40 samples from human chromosome 18.

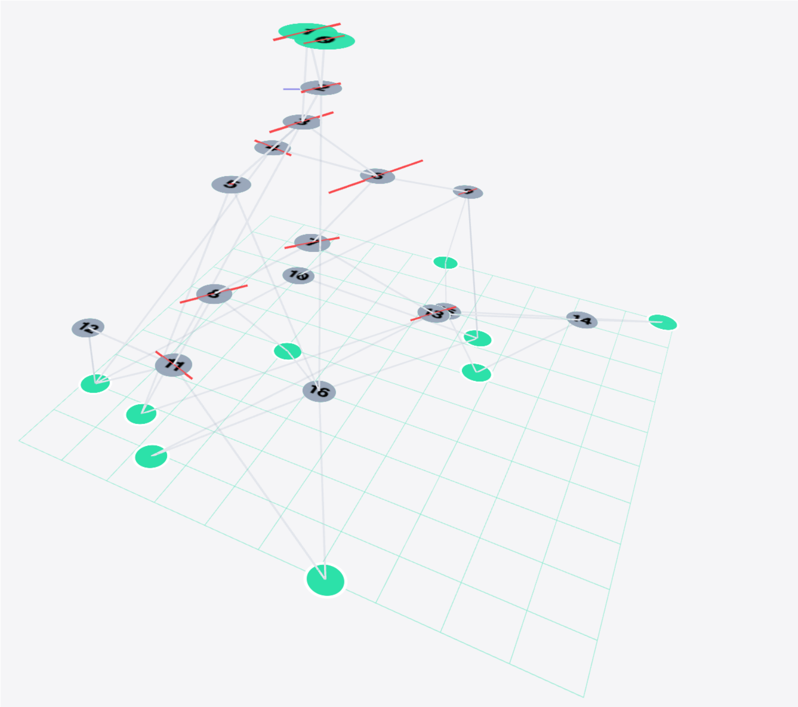

Figure S3: A ‘spatial diff’ view, comparing location inference between continuous FastGaia and Wohns midpoint algorithms, applied to a tree sequence simulated in SLiM.
